# Reliable and cost-efficient protection scheme for 5G fronthaul/backhaul network

**DOI:** 10.1016/j.heliyon.2023.e14215

**Published:** 2023-03-02

**Authors:** Syed Saeed Jaffer, Ashiq Hussain, Muhammad Ali Qureshi, Yousaf Khan, Jawad Mirza, Khurram Karim Qureshi, Muhammad Mahmood Ali

**Affiliations:** a Department of Industrial Electronics Engineering, Institute of Industrial Electronics Engineering (IIEE), PCSIR, Karachi, Pakistan; b Department of Electrical Engineering, Air University, Islamabad, Pakistan; c Department of Information & Communication Engineering, The Islamia University of Bahawalpur, Pakistan; d Department of Electrical Engineering, UET, Peshawar, Pakistan; e SEECS Photonics Research Group, Islamabad, Pakistan; f Department of Electrical Engineering and Center for Communication Systems and Sensing, King Fahd University of Petroleum & Minerals, Dhahran, Saudi Arabia; g Centre for Mathematical Modelling and Intelligent Systems for Health and Environment (MISHE), Atlantic Technological University Sligo, Ash Lane, F91 YW50 Sligo, Ireland; h Department of Mechatronic Engineering, Atlantic Technological University Sligo, Ash Lane, F91 YW50 Sligo, Ireland

**Keywords:** Centralized radio access network (C-RAN), Free space optics (FSO), Passive optical network (PON), Fronthaul, 5G, UDWDM, CAPEX, Reliability

## Abstract

One of the challenges for Mobile Network Operators (MNO) in 5G deployment is to ensure reliability in the various sections of the network as the new services and applications, for instance, video on demand, telemedicine, online learning, smart transportation and augmented reality require not only high bandwidth but also demand uninterrupted service. However, this reliability requires substantial investment. Therefore, MNO only deploys protection or backup resources in the network unless it is cost-effective. The study aims to present a reliable and low-cost protection scheme based on an Ultra-Dense Wavelength Division Multiplexing Passive Optical Network (UDWDM PON) for the transport layer of the 5G network, i.e., for the fronthaul/backhaul section. We have evaluated the Capital Expenditure (CAPEX) cost of UDWDM PON with and without protection in a dense urban area. We also measure the figure of merit between the cost and reliability of the system and, subsequently, confirm that the proposed protection scheme can achieve system reliability up to four nines with very low additional CAPEX investment. Finally, the efficacy of the proposed protection scheme is also demonstrated through simulation experiments.

## 1 Introduction

Mobile networks are being evolved or upgraded due to the advances in wireless devices and the demands of various industrial applications. Cellular users dream of bandwidth-consuming applications (more Netflix and YouTube streaming). In contrast, on the eve of the fourth industrial revolution, the industry turned into a vertical market (data-driven industry) in which each business sector has its bandwidth appetite based on faster and more reliable data delivery. The author [1] reported that the data would be the key enabler or the driving force for the fourth-generation industrial revolution. As a result, global internet traffic will be compounded every year. One of the primary requirements of industrial users, as well as residential (partially) users, is the always “live” network because they are more dependable on technology, i.e., the user or clients requires a 24/7 available network on which they exchange data with reliability. To meet the expectations envisioned by the client in terms of data transmission, the wireless networks based on 5G are supposed to be the front-end communication network to support the vertical industry demands as the existing cellular network is almost approaching the Shannon limit (the 4G networks are not substantial enough to support massively connected devices, i.e., 50 billion ubiquitous connected devices with low latency and high spectral efficiency, which will be crucial for the future communication and computing).^1^

The 5G network designers are committed to supporting high data transmission rates >10 Gb/s for residential clients, i.e., the wireless users experience enhanced mobile broadband service in the line of sight (LOS) condition, whereas, in non-LOS (stationary and mobility condition) the data rate is set to 3 Gb/s and 100 Mb/s respectively [2]. The 5G researchers must introduce novel techniques in the entire network layer to achieve these goals and standards. Therefore, software-defined networking (SDN), network function virtualization (NFV), non-orthogonal multiple access (NOMA), advanced modulation schemes, cognitive radio, the adaptation of multiple-input multiple-output (MIMO) antennas technology and network slicing would be the basic building blocks of 5G network.

Apart from that, the flexible and scalable architecture is also required in the radio access network (RAN) layer, as in 5G million of devices have to be accommodated in the small location, that requires the picocells, femtocells, and microcells or we can say that the future 5G RAN would be based on the heterogeneous network. To accommodate such a number of users and their traffic density, the 5G operators requires a huge investment in the access layer, which challenges MNO, particularly in terms of capital and operational cost as identified by the researchers in [3], [4]. Therefore, the 5G operators will require the cloud functionalities in RAN, called the centralized radio access network (CRAN), to protect their investment, where all or some of the functions of the baseband units are migrated from the base transceiver station to the cloud. However, the CRAN requires a fiber-based network in the access layer and in the transport layer of 5G, so future 5G networks would be based on hybrid technology that includes both optical and wireless networks [3], [5].

Passive Optical Networks (PONs) are believed to be the best technology for deploying broadband access networks as it is considered the best fiber-sharing technology among multiple users. However, the PON without protection scheme is considered very vulnerable against always live network approach as the breakdown of fiber in the feeder or distribution section can disrupt the network service. As we have indicated earlier, the 5G clients/end users require reliability or availability of the network, 24/7, especially the business client. On the other hand, the MNO provides reliability in the network when its capital investment is aligned with its revenues. Therefore, this paper proposes a reliable and low-cost protection scheme for the 5G fronthaul/backhaul network based on UDWDM- PON network.

The rest of the paper is arranged as follows. Section 2 describes the basic architecture of PON along with the challenges that the PON industry has to face to support the 5G expectations. Section 3 discusses the architecture of WDM-based PON with and without protection. Section 4 elucidates the cost MNO incurs due to the addition of protection or backup resources in the transport layer to meet the demands of reliability or availability of 5G standards. We will also discuss the reliability analysis of the protection scheme in Section 4. While in Section 5, we will discuss the performance analysis and the power budget analysis of the proposed protection scheme. Finally, Section 6 concludes the paper.

## 2 Passive optical network

**Figure 1:**
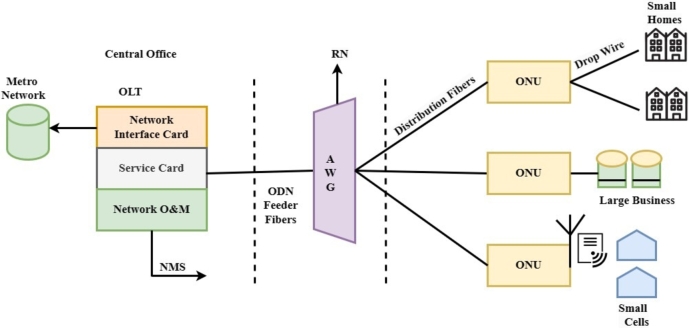
A basic architecture of passive optical network.

PON is a Point to Multipoint (PtMP) technology that provides fiber connectivity to end-users on a sharing basis. The basic architecture of PON, illustrated in Fig. 1, is very simple and consists of only three elements, or we can say that PON is three-layered network architecture arranged in a tree topology (this simple architecture, we referred in this paper, standalone PON system). The head end is called the optical line terminal (OLT), mostly installed at the service provider's central office, transmitting data towards the subscriber equipment called optical network unit (ONU), usually at 1310 nm, 1550 nm or 1610 nm optical window. The network management center (NMS) is also installed in the central office for the whole network's operation and maintenance (O& M). A single OLT can serve several ONUs through the splitter up to 20 km (standard range).

In contrast, the function of an ONU placed either at indoor or outdoor locations is intended to serve a large number of residential or corporate users. The ONU converts the optical signal into the electrical signal and delivers many broadband services, such as voice over internet protocol, plain old telephony service, etc., to end-users. The physical connection between OLT and ONU is based on a fiber infrastructure called an optical distribution network (ODN). The ODN comprises an optical fiber and passive splitter or array waveguide grating (AWG), which separates or routes the wavelength to the respective ONU. The fiber that runs between OLT and the splitter is called feeder fiber, and the remaining fiber section that terminates between the splitter and ONUs is called the distribution fiber.

The PON was initially meant to design for the fixed broadband service to replace the traditional copper cable from the optical fiber in the access part of the public switch telecommunication network (PSTN). Therefore, many PSTN service providers started to deploy PON to fulfill their customer bandwidth demands. The idea of sharing fiber infrastructure among multiple PSTN users through PON was originally conceived by a full-service access network (FSAN) in the early 90's. Since then Institute of Electrical and Electronics Engineering (IEEE) and the International Telecommunication Union (ITU) have set the PON standard [6], [7], [8]. Over the last two decades, different PON technologies have been standardized, whereas some new standards of PON are expected to be deployed in the coming years as discussed in [9], [10], [11]. In each advanced standard, increasing transmission rates both in downlink and uplink directions with extended reach and split ratios remain the point of focus of the PON industry.

The PON requires a multiple access technique for data transmission among the number of connected nodes. Depending on the transmission technique, PON is classified into two categories: time division multiplexing (TDM) and wavelength division multiplexing (WDM). In TDM-PON, the OLT broadcasts the signal in the downlink channel in a different recurring time slot on a single wavelength. In contrast, the ONU uses a different wavelength for signal transmission in the uplink channel. In WDM-PON, the OLT and ONU transmit their data on different sets of wavelengths or their assigned wavelengths. Both technologies have their merits and demerits and are summarized by the researchers in [9], [10], [11]. The TDM-PON approach is currently sufficient to meet fixed residential or fiber-to-the-home (FTTH) demands. However, the PON industry is now rethinking supporting the fiber to antenna (FTTA) application. Therefore, the FSAN introduced the partial or complete WDM approach. The current TDM approach is quite inefficient in supporting 5G fronthaul/backhaul service, as it offers higher bandwidth and longer reach compared to the TDM approach [12][13]. However, the partial or hybrid TWDM multiplexing approach [14] or alternative schemes such as OFDM [15] do not furnish a dedicated wavelength to each user besides its low-cost transceiver and backward compatibility to ODN. Whereas, the ultra-dense wavelength division multiplexing (UDWDM) PON offers the lambda to the user approach while maintaining channel spacing at a few GHz [16], thus allowing a scalable PON with increased numbers of users [17].

Furthermore, the UDWDM-PON is developed under the EU FP7 (COCONUT) project [18], [19], employing coherent detection, excellent wavelength selectivity, high sensitivity, long reach and high splitting ratios. Therefore, we have proposed the UDWDM-PON as a new emerging cellular application, i.e., for the 5G transport services. In the next sub-section, we first identified the 5G transport requirements, followed by a discussion on the suitability of PON in terms of the 5G transport context.

### 2.1 5G transport requirements

The new 5G RAN service redefined the transport requirements of 5G as discussed in [20]. The CRAN decouples the RAN functionalities, i.e., the remote radio head (RRH) and baseband unit (BBU), in terms of physical locations, such that the CRAN moves the completely or partially BBU functionalities towards the cloud. The physical distance between the RRH and BBU maybe thousands of meters; therefore, the transport network is the mandatory requirement of CRAN [21]. The bandwidth and latency requirements of the CRAN transport section, depending on the split option and the BBU functionalities (in or out of the cell location), are summarized by the author in [3][20]. In Table 1, the authors [22][23], [24], [25] show the bandwidth and latency requirements of 8 potential split options identified by the 3rd generation partnership project (3GPP). Each split option has its merits and demerits; therefore, the choice of choosing the CRAN split option depends upon the operator's business model or their customized solution. The PON base transport network can meet the demands of CRAN split options 6 and 7 (also called the non-ideal fronthaul solution). Therefore, in the following subsection, we will discuss the challenges of the PON in the context of CRAN split options, i.e., 6 and 7.

**Table 1:** Data calculate on the basis of 100 MHZ, 256 QAM and 08 MIMO configuration.

Split option	Uplink Bandwidth	Downlink Bandwidth	Latency
1	2000 Mb/s	3000 Mb/s	10 ms

2	2016 Mb/s	3024 Mb/s	10 ms

3	2048 MB/s	4000 MB/s	10-100 μs
4	3000 Mb/s	5000 Mb/s	
5	4000 Mb/s	5000 Mb/s	
6	4133 Mb/s	5640 Mb/s	

7(a)	10-22 Gb/s	16-21 Gb/s	1-10 ms
7(b)	37-86 Gb/s	53-86 Gb/s	
7(c)	10-22 Gb/s	53-86 Gb/s	

8(CPRI)	157.3 Gb/s	157.3 Gb/s	1 ms

### 2.2 Adaptation of PON for 5G transport solution

Recognizing the role of PON for 5G transport, the PON industry started deliberation over the cost, complexity, and timeline issue of the new generation of PON for the transmission of 5G signal over PON media. The biggest requirement of PON is how to reuse the already deployed passive ODN for 5G services since the laying, digging, and trenching of fiber require almost 70% of the total PON capital expenditure (CAPEX) as referred by [12], [4]. Therefore, the next generation of PON should be compatible with the existing fiber infrastructure of PON ODN. The other requirement of PON to support 5G services is improved power budgeting, i.e., the next generation of PON requires extended reach and split ratio to transmit data towards multiple small cell locations. Another important aspect that needs to be addressed is the design of a power-efficient modulation format for PON, as the high data rate can create nonlinearity issues in fiber transmission.

The PON also requires low latency service as the various 5G services are delay sensitive. In 5G, the end-to-end latency requirement can be below 1 ms, which in the traditional PON scheme is difficult to maintain. Therefore, an improved bandwidth allocation method is required in PON transmission to support 5G delay-sensitive applications. In addition to the above, the other challenge for the PON industry is synchronization, which contributes due to the signal propagation over fiber with different delays. Therefore, precision timing is also the key concern in the next generation of PON. To cope with the mentioned challenges, narrow-spacing WDM PON is an ideal PON technology for a 5G transport solution. In the next section, we will discuss the system architecture of UDWDM PON.

## 3 UDWDM PON architecture

**Figure 2:**
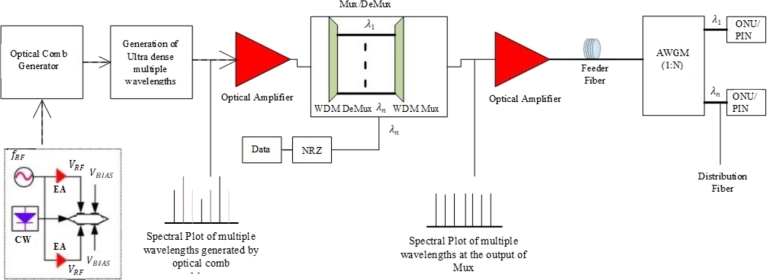
Standalone UDWDM PON system.

The basic architecture of UDWDM PON is shown in Fig. 2, in which the OLT is installed at the central office of the telecom building and ONU at the cell site location or small cells. Whereas the AWG is housed at the remote node (RN), the intermediate site between the OLT and ONU, used to split the bunch of wavelengths and distribute them towards the ONU through the distribution Fiber (DF). In UDWDM-PON, generally, the series of discrete spectral components (frequency combs) with narrow spacing are generated through an optical comb generator that consists of a single continuous wave (CW) laser diode driven by an external sinusoidal RF source, an electrical amplifier (EA), and a dual-drive Mach-Zehnder modulator (DD-MZM). One of the major drawbacks of the UDWDM PON-based system is that it has very low reliability because there is no protection feature available in the overall system, especially in the feeder or the distribution segment of the network. Therefore, in the next section, we will first discuss the already available protection schemes in the literature for PON. Then we will propose a reliable and cost-efficient protection scheme for UDWDM PON. Adding a protection feature in the UDWDM PON makes it a potential candidate technology for the 5G fronthaul/backhaul service.

### 3.1 Evolution of protection scheme in PON

It is proven that the unprotected PON is highly unreliable, as indicated by researchers [26], [27]. Therefore, it is important to study the protection mechanism of PON. In literature, several protection schemes for different PON standards are discussed [28], [29], [30] in which the various protection schemes applied to PON's standard are compared in terms of the number/type of components, fiber length, connection availability, failure impact factor and power consumption. The PON protection architecture started through ITU-T G.983.1 documentations of types A, B, C, and D [31], [32], [33], [34], [35]. The ITU Telecom section referred to these protection schemes as partially or fully protection schemes. The partial protection scheme is referred to as types A and B. In contrast, a fully costly protection scheme is referred to as type C and D, providing end-to-end reliability or full duplication to each part of the network entity. The main consideration in a partial protection scheme is based on the cost factor since the protection cost is considered in CAPEX cost. Therefore, the operator deployed the PON protection scheme after the detailed CAPEX cost analysis.

Our approach, in this manuscript, is based on a partially protection scheme, and it is designed by considering the following assumptions, i.e., to secure a large number of users from the outage, reduce the cost incurred to provide protection and the failure impact robustness (FIR) of the network. In the proposed PON model, the critical elements of the network are OLT, ODN, RN, and ONU. However, protecting each crucial element of the network is not preferable or cost-effective. Therefore, we will ensure the reliability of those network elements more prone to fault and ignore the reliability of those elements with low failure in time (FIT) or mean time to repair (MTTR) value. In PON, it is studied that in-house components have a low fault ratio and MTTR value compared to on-field equipment.

Moreover, the author in [36] reported that the federal communication commission estimate that, in an optical network, nearly 50% fault occurs due to the disruption in the fiber link. Therefore, we have identified the Feeder Fiber (FF) and Distribution Fiber (DF) sections that require more attention than other indoor elements. Typical values of the FIT and MTTR of various system components are reported in Table 2 [37], [38], [28], [39].

**Table 2:** Component FIT & MTTR values [37], [38], [28], [39].

Component	Failure Rate (FIT)	Mean Time to Repair (MTTR)
OLT	256	2
ONU	256	2
EDFA	200	2
UDWDM	7200	2
Fiber/km	60	4
AWG	2400	2
Optical switch	200	2
Mux	2400	2
DEMUX	2400	2
FSO atmospheric turbulence	595	2
FSO Transmitter	500	2
FSO Receiver	100	2

The FIT is a unit for measuring the failure rates, where one FIT means one failure in 10^9^ h. Generally, the optical path is protected with the 1+1 or 1:1 techniques. Therefore, in literature, the authors [40] presented the idea of deploying fully protected ODN with duplicated fiber. The suggested architecture is very effective against any interruption. However, due to the installation of new fiber routes, these schemes are very costly. In other work, for the DF path, the authors [41], [42] suggested that the ONU at the customer end can be protected with the neighboring ONU via new interconnecting fiber routes. However, this scheme also requires the burying of new fiber routes between two ONUs which can be cumbersome, especially in dense urban areas. This paper presents for the first time an end-to-end ODN-based protection scheme, for the UDWDM PON system based on a partially fiber approach, for the fronthaul/backhaul service for 5G users in a dense urban area (subscriber >2048/km^2^). We have deployed the strategy of bidirectional ring fiber to protect the FF routes while employing free space optics (FSO) links for protecting the DF path. In the next sub-section, we will first discuss the basics of FSO, and subsequently, in the next sub-section, we will present the proposed protection architecture for the UDWDM PON.

### 3.2 FSO wireless technology

Free Space Optics (FSO) is a wireless optical technology that offers a high data rate over a short distance nearly equal to fiber-based optical communication. Whereas, the installation cost of an FSO-based optical link is very low as compared to a fiber-based optical link because an FSO link is established with the pair of telescopes that are installed at the transmitter and receiver section of the communication link [43], [5]. The other advantage of FSO, which makes it ideal for fronthaul technology, is having a license-free spectrum with no electromagnetic interference as FSO operates in the optical region of the electromagnetic spectrum [43]. The FSO systems typically work in the LOS scenario; however, the same can work in the NLOS scenario by employing relay-assisted FSO systems [44]. Therefore, the FSO links are widely adopted as protection paths for passive optical networks, optical data centers, and 5G/6G front hauls [5], [6]. In our design, the major motivation behind the adaptation of the FSO link at DF link, it offers numerous advantages over physical fiber deployment, which the authors also report in [5], [45], i.e., the FSO provides licensed free operation with the added feature of flexibility in the deployment cycle. Therefore, the FSO link at the DF can decrease the overall system CAPEX and OPEX cost [4]. Besides the above, PON and FSO operate in the same transmission window, therefore, the data can switch from PON to the FSO link transparently.

However, FSO systems have various challenges which affect the overall system performance. These include weather-induced disturbance (atmospheric attenuation and turbulence), geometrical losses, misalignment of transmitter and receiver telescopes i.e. the line of sight blockage (LOS) [46]. Two types of LOS blockage affect the FSO communication [4]. The first one is permanent LOS blockage occurs due to the presence of trees or buildings, etc., which permanently blocks the optical beam from reaching the receiver's telescope. The second type is partial LOS blockage occurs due to the momentary presence of flying objects like birds, drones, etc. However, the major impairment affecting the system's performance is atmospheric attenuation or turbulence [46].

In FSO transmission, atmospheric turbulence, which mainly occurs due to variation in the temperature and pressure of the atmosphere (also called intensity scintillation), is considered one of the primary contributors that degrade the FSO system performance. So in literature, several statistical modeling techniques have been discussed that can predict and mitigate the effect of channel degradation occurs due to intensity scintillation [46]. Some general statistical models are lognormal, K distribution, negative exponential, gamma-gamma, and lognormal Rice model [46]. These statistical modeling techniques are used for different atmospheric turbulence, such as for weak turbulence or in clear sky weather the lognormal distribution is suitable as pointed out by the researcher [43]. The K distribution is generally characterized for modeling the moderate turbulence over several kilometers [43]. The negative exponential is used to model the severely deteriorated atmospheric channel [46], whereas the gamma-gamma distribution is the generalized model that is applied in various turbulence conditions [46]. In the gamma-gamma distribution, the intensity of the channel *I* is the function of both large and small atmospheric effects [46]. Both large and small fluctuations follow a gamma-gamma distribution, their value typically varies from $10^{-12}$ to $10^{-17}$ (weak to strong turbulence) [46].

In our design, the FSO transmitting and receiving telescopes are installed at heights like rooftops or towers, etc, therefore, the LOS can be easily established between remote nodes and ONUs so permanent LOS blockage, in our case, is not possible. While partial LOS blockage (due to various flying objects like birds, drones, etc.) is less likely, therefore, we have ignored its effect and the only effect we considered in the simulation is the beam spreading or the intensity scintillation.

### 3.3 Proposed protection architecture

**Figure 3:**
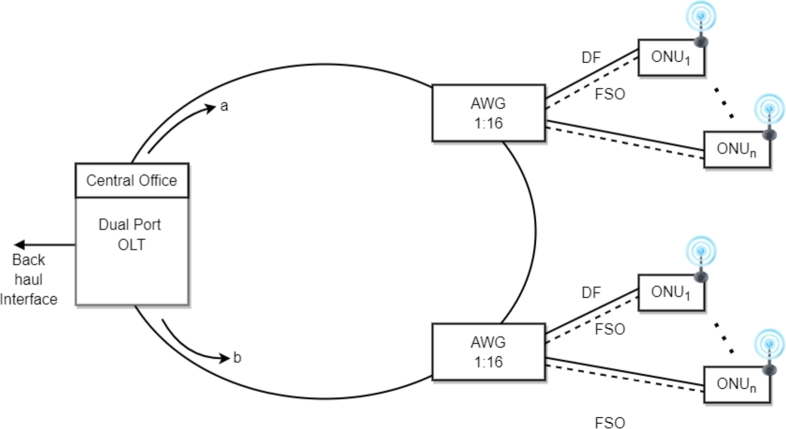
Optical-wireless based protection architecture with single bidirectional fiber ring topology.

The proposed protection scheme proposed in this work, called optical wireless base protection architecture with single bidirectional fiber ring topology, ensures ubiquitous connectivity for the ODN segment of PON. The proposed protection scheme is also shown in Fig. 3. We have deployed the protection path both at the FF level and in the DF by considering the value of FIT and MTTR indicated in Table 2. In Section 3, we have already discussed the working of UDWDM PON in a normal mode of operation. The bidirectional single fiber ring topology in protection architecture is deployed to protect the FF section. The dual fiber ring architecture can also be used to protect the FF. However, this approach is very costly because the system requires non-standard equipment at each ONU, which can elevate the cost of protection. The dual fiber ring is mostly used in the core and metro network (PDH, SDH), whereas in an access network, which is cost-sensitive, usually deployed with single-ring fiber. At the FF section, in the normal mode of operation, the OLT transmits data, for instance, through the path “a” clockwise or in the forward direction, but in case of unavailability of an active link, the OLT starts the transmission in the reverse direction by the following path “b.” To provide the configuration in the network, the operator requires only dual-ports OLT and bidirectional ring, whereas, to switch or control the direction of the optical signal, the media access control (MAC) protocol is used without any additional control system.

At the DF section, as shown in Fig. 3, in the regular mode of operation, the optical signal propagates from AWG to ONU through DF, but in case of the failure of DF (due to fiber cut or maintenance work), the optical switch will divert the traffic from the distribution fiber to the protection path, i.e., on the FSO link. To redirect the traffic of ONU from DF to FSO, an optical switch will be installed at the ONU, which switches the traffic on the working or protection path by sending a control signal (CS) at the RN. If the status of CS=1, AWG transmits data on the DF path, and when CS = 0, AWG switches the traffic on the FSO link. We have also proposed the narrow beam FSO link for data transmission because it decreases the FSO link geometric loss, increases the FSO link length and improves security features in the system. Table 3 gives a qualitative comparison of different protection schemes widely adopted in the PON and the proposed protection scheme.

**Table 3:** Comparison of proposed optical wireless-based protection architecture (single bi-directional fiber ring topology with existing PON protection schemes.

Parameters	Partially Protection (Type A/B)	Fully Protection (Type C/D)	Ring-based (Single Fiber)	Ring-based (Double Fiber)	Hybrid Protection (Ring-Star-Ring)	Proposed
Protection level	FF	Entire PON	FF	FF	ODN	ODN
Availability	Low	High	Low	High	High	High
Reliability	Low	Low	Low	High	Moderate	Moderate
CAPEX Cost	Low	High	Low	High	High	Very Low
Fault restoration time	High	High	Moderate	Moderate	High	Low
Deployment time	High	High	High	High	High	Very Low
Flexibility	Low	Low	Low	Low	Low	Very High
Ref.	[47]	[48]	[49]	[50]	[51]	**Proposed**

## 4 Network analysis

Any protection method is normally evaluated by two critical criteria, the availability of the protection link and the cost bearing by the operators to provide the protection features in the network [37]. Therefore, in this section, first, we evaluate the reliability analysis of the system and, secondly, the CAPEX cost analysis of the proposed protection scheme.

### 4.1 Reliability analysis

In the telecommunication industry, unavailability (downtime) is the probability that any network component, system, or subsystem is not working for a specific duration of time, whereas system availability (uptime) is the probabilistic measure of the duration of time when the system is working. The system availability is calculated in percentage (%); for instance, if a system has the availability of 99.999%, it means that the system downtime is very short or less than 5 minutes in a year, while if the downtime starts increasing, it means system unavailability time is getting high.

**Figure 4:**
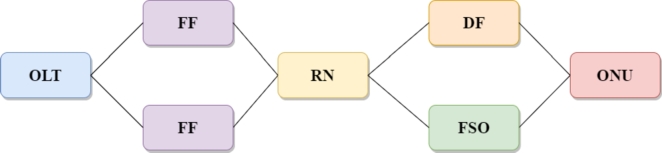
Block reliability model of the proposed architecture.

The block reliability model of the proposed protected architecture is shown in Fig. 4. The key parts of the system are OLT, which contains the optical transceiver, and optical/electrical chips. The other key component in the system is ONU which also includes the Laser, optical detector, and electrical chips. The failure analysis of both OLT and ONU is almost the same as the failure in the components of both OLT and ONU occurs for the same reasons. The first breakdown in the components is the value of Laser wear out, which is 10 years up to 40 °C, and the second reason is the random failure rates of individual components of OLT/ONU, i.e., Laser, receiver and electrical chips. The other important components in the system are AWG, optical switch and Mux/Demux; their failure analysis is based on the random failure of the overall component. In comparison, the failure rate of fiber in an urban area is considered based on per km. To measure the reliability analysis of the FSO link (direct link), we have included the failure rate due to the equipment and environmental turbulence failure. In the literature, it is reported that the FSO system availability is more than 99.99% for the link length of 1 km in clear sky conditions.

The total unavailability of the proposed network architecture ($U_{N}$) is calculated through Eq. (1) [52]. After adding protection features in the system, it would be regarded as a combination of parallel and series systems. The unreliability of the parallel and series components can be calculated through Eq. (2) [53] and Eq. (3) [54].

(1) $$\text{System Unavailability }(U_{N})=(1-\text{availability})\times 365\times 24\times 60$$

(2) $$U_{parallel}=\prod_{i=1}^{n}U_{i}$$

(3) $$U_{series}=\sum_{i=1}^{n}U_{i}$$

whereas, in Eq. (4) [54], the unavailability of the component ($U_{i}$) can be estimated through MTTR and FIT, whereas the value of MTTR is the average time required to repair the device. The value of MTTR and FIT of all the components used in the model are reported in Table 2, while the component unavailability time is obtained from Eq. (4) reported in Table 4 [55], [38].

(4) $$U_{i}=\text{MTTR}\times \text{FIT}\times 10^{-9}$$

The value of ($U_{N}$) for our proposed architecture can be obtained through Eq. (5).

(5) $$U_{N}=U_{UDWDM}+U_{MUX}+U_{DEMUX}+U_{OLT}+U_{EDFA_{1}}+{\left(U_{FF}\right)}^{2}+U_{AWG}+U_{EDFA_{2}}+U_{OS}+(U_{DF}\times U_{FSO_{T}})+U_{ONU}$$

where

(6) $$U_{FF}=d_{1}\times U_{F},\;\text{where}\;d_{1}=10\;\text{km}$$

(7) $$U_{DF}=d_{2}\times U_{F},\;\text{where}\;d_{2}=1\;\text{km}$$

(8) $$U_{FSO_{T}}=U_{FSO_{Tur}}+U_{FSO_{Rx}}+U_{FSO_{T_{x}}}$$

**Table 4:** Component & Link-wise Unavailability data [38], [55].

Component	Symbol	Unavailability Time/10^9^
OLT	*U*_*OLT*_	5.12 × 10^−7^
ONU	*U*_*ONU*_	5.12 × 10^−7^
EDFA	*U*_*EDFA*_	4 × 10^−7^
UDWDM	*U*_*UDWDM*_	1.44 × 10^−5^
Fiber	*U*_*F*_	2.4 × 10^−7^
AWG	*U*_*AWG*_	4.8 × 10^−6^
Optical switch	*U*_*OS*_	4 × 10^−7^
Mux	*U*_*MUX*_	4.8 × 10^−6^
DEMUX	*U*_*DEMUX*_	4.8 × 10^−6^
FSO (Equipment + Turbulence)	$U_{FSO_{T}}$	8.29 × 10^−9^
FSO Turbulence	$U_{FSO_{Tur}}$	1.19 × 10^−6^
FSO Transmitter	$U_{FSO_{Tx}}$	1 × 10^−6^
FSO Receiver	$U_{FSO_{Rx}}$	2 × 10^−7^

From Eqs. (6), (7), and (8), the unavailability of the overall system, $U_{N}$, can be quantitatively expressed as Eq. (9):

(9) $$U_{N}=3.22322\times 10^{-5}$$

The value of *R* suggests that the network has high reliability, up to four nines, i.e., 99.9946%. Furthermore, Table 5 mentions the notion of time of availability vs. system downtime.

**Table 5:** The Link availability of Five Nines.

Availability (%)	Downtime per year	Downtime per month	Downtime per week
One Nine (90%)	36.5 days	72 hrs	16.8 hrs
Two Nines (99%)	3.65 days	7.2 hrs	1.68 hrs
Three Nines (99.9%)	8.76 hours	43.8 min	10.1 min
Four Nines (99.99%)	52.56 min	4.32 min	1.01 min
Five Nines (99.999%)	5.26 min	25.9 sec	6.05 sec

So the reliability of the network, *R*, can be calculated through Eq. (10).

(10) $$R=1-U_{N}=99.9967\text{\%}$$

### 4.2 CAPEX analysis of unprotected and protected scheme

Here, we evaluate and compare the CAPEX cost of unprotected and protected UDWDM PON for the fronthaul/backhaul service. Since the cost of providing protection is reported in the CAPEX, we will study only the CAPEX cost of the model with and without protection. In the next section, we will first identify the factors that affect the CAPEX cost and then compute the numeric cost of all network components and physical structure, i.e., the cost of the OLT/ONU/RN component, fiber digging/trenching, and FSO modules.

#### 4.2.1 Factor affecting CAPEX cost

The estimation of the CAPEX cost of a communication system is an important task. It includes the costs of equipment, network planning, site acquisition, transportation cost of the equipment, labor cost, site rental fee and licensing fee etc. All above costs also vary on a regional basis, so we have assumed all the equipment costs and engineer/labor charges as per the average price available in the subcontinent (South Asia); therefore, these costs apply to Pakistan, Bangladesh, and India.

#### 4.2.2 CAPEX cost of unprotected UDWDM PON

We will compute the CAPEX cost of unprotected architecture for 32 small cells in a dense urban area. We have assumed that the feeder fiber length, i.e., from OLT to RN, is up to 10 km and the length of distribution fiber from RN to ONU is a maximum of up to 1 km. The cost of the unprotected model, shown in Fig. 3, would be calculated through the Eq. (11), whereas the numeric cost of the components and the physical structure is provided in Table 6 [56], [57]. The modules or hardware inventory costs used in Table 6 are based on the commercial prices available on online resources and the well-known local vendors. However, if available, fiber prices may change according to the specific network operator's data.

(11) $$C_{U}=C_{UDWDM}+2\times C_{EDFA}+C_{MUX}+C_{DeMux}+C_{OLT}+C_{FF}+C_{AWG}+C_{DF}+32\times C_{ONU}+C_{INS}$$

where $C_{UDWDM}$, $C_{EDFA}$, $C_{MUX}$, $C_{DeMUX}$ and $C_{AWG}$ are the cost of EDFA amplifier, Mux, DeMux, and AWG respectively. The $C_{OLT}$ represents the cost of OLT, which depends on the OLT's reach and splitting ratio. The $C_{ONU}$ is the cost of ONU, which is the function of the types of the transceiver used in the ONUs. The $C_{FF}$ and $C_{DF}$ are the cost of FF and DF. We have assumed that the cost of fiber is constant, which means this cost is independent of the fiber types. FF and DF costs are proportional to the deployment distances and the cost of trenching and laying the fibers. The $C_{INS}$ is the total installation cost incurred to provide fronthaul/backhaul connection to 32 small cells in the dense urban area that depends upon the number of engineers/technicians and their salary rate. The description of the labor cost involved in the CAPEX is also reported in Table 7.

**Table 6:** CAPEX cost of unprotected architecture (vlaues were acquired from www.OEQUEST.com).

Cost Factor	Component	Description	Nos.	Unit cost ($)	Total Cost ($)
Equipment	OLT	Include OLT Housing/Chassis cost, CW Laser, MZM	1	$4,000.00	$4,000.00
	EDFA	Signal amplification	2	$180.00	$360.00
	MUX	1:32	1	$330.00	$330.00
	De-MUX	32	1	$330.00	$330.00
	AWG	1:16 (Housing / chassis cost with thermal packaging)	2	$400.00	$800.00
	ONU	with tunable laser and filter	32	$150.00	$4,800.00
	Total Equipment cost:				$**10,620.00**

Fiber	Feeder fiber	length of fiber required at FF section	10 km	$80.00 / km	$800.00
	Distribution fiber	length of fiber at DF section	(32 × 0.5) average length of a single DF is taken as 0.5 km = 16 km	$80.00 / km	$1,280.00
	Total Fiber cost:				$**2,080.00**

Installation	Cost of installation of all equipment	central office equipment / remote location installation / ONU	08 engineers required for 20 days	$15.00 / day	$2,400.00
	cost of installation of fiber	digging / trenching / pumping / splicing of FF & DF	10 km + 16 km = 26 km	$3,000.00 / km	$78,000.00
	Total Installation cost:				$80,400.00

	**TOTAL COST:**				$**93**,**100.00**

**Table 7:** Description of labor (installation) cost variables used in this paper (these costs are taken through the reference of well-known practices by the local vendors).

Installation Heads	Details
*C*_*INS*−1_	Cost of installation at the head-end or central building (telecom office)
*C*_*INS*−2_	Tower installation cost (roof-top tower)
*C*_*INS*−3_	Installation cost incurred due to the deployment of light post
*C*_*INS*−4_	Cost incurred due to FSO link
*C*_*INS*−5_	Deployment cost of optical network unit
*C*_*INS*−6_	Cost incurred due to AWG modules
*C*_*INS*−7_	Deployment cost of RRH modules
*C*_*INS*−8_	Splicing cost
*C*_*INS*−9_	Installation cost due to fiber deployment at feeder fiber section
*C*_*INS*−10_	Fiber laying & trenching costs incurred at the distribution ends

#### 4.2.3 CAPEX cost of protected UDWDM PON

The architecture of the proposed protection scheme is shown in Fig. 3. The mathematical description of the CAPEX cost of the protected architecture, i.e., $C_{P}$, can be computed through the following Eq. (12). The extra components required to deploy the protection feature in the architecture are the dual-port OLT, which supports the ring architecture in the FF section. In case of the failure of one signal path in the ring transmission, the dual port OLT senses this anomaly in the system and starts transmission in the reverse direction. The other extra protection component used in the system is the FSO transceiver, which depends upon the FSO link length and the operating wavelength of the FSO link. The optical switch is also used in the system to switch the transmission of DF in case of a faulty FSO link. The costs of dual-port OLT, FSO transceiver and optical switch are also mentioned separately in Table 8 [56], [57], while the installation cost of the FSO link is also added to the installation cost.^2^

(12) $$C_{P}=C_{UDWDM}+2\times C_{EDFA}+C_{MUX}+C_{DEMUX}+C_{D-OLT}+C_{FF}+C_{AWG}+C_{DF}+32\times C_{ONU}+32\times C_{FSO}+C_{INS}$$

**Table 8:** CAPEX cost of protected architecture (vlaues were acquired from www.OEQUEST.com).

Cost Factor	Component	Description	Nos.	Unit cost ($)	Total Cost ($)
Equipment	Dual-Port OLT	Include OLD Housing/Chassis cost, CW Laser, MZM	1	$5,000.00	$5,000.00
	EDFA	Signal amplification	2	180	$360.00
	MUX	1:32	1	$330.00	$330.00
	De-MUX	32	1	$330.00	$330.00
	Optical Switch	Sensing and diverting DF traffic	1	$50.00	$50.00
	AWG	1:16 (Housing / chassis cost with thermal packaging)	2	$400.00	$800.00
	FSO transceiver	Operating at 1550 nm wavelength for 1 km range	32	$400.00	$12,800.00
	FSO Mounting panel	Mounting panel	32	$10.00	$320.00
	ONU	with tunable laser and filter	32	$150.00	$4,800.00
	Total Equipment cost:				$**24,790.00**

Fiber	Feeder fiber	length of fiber required at FF section	10 km	$80.00 / km	$800.00
	Distribution fiber	length of fiber at DF section	(32 × 0.5) average length of a single DF is taken as 0.5 km = 16 km	$80.00 / km	$1,280.00
	Total Fiber cost:				$**2,080.00**

Installation	Cost of installation of all equipment	central office equipment / remote location installation / ONU / FSO link	10 engineers required for 20 days	$15.00 / day	$3,000.00
	cost of installation of fiber	digging / trenching / pumping / splicing of FF & DF	26 km	$3,000.00 / km	$78,000.00
	Total Installation cost:				$81,000.00

	**TOTAL COST:**				$**107**,**870.00**

**Figure 5:**
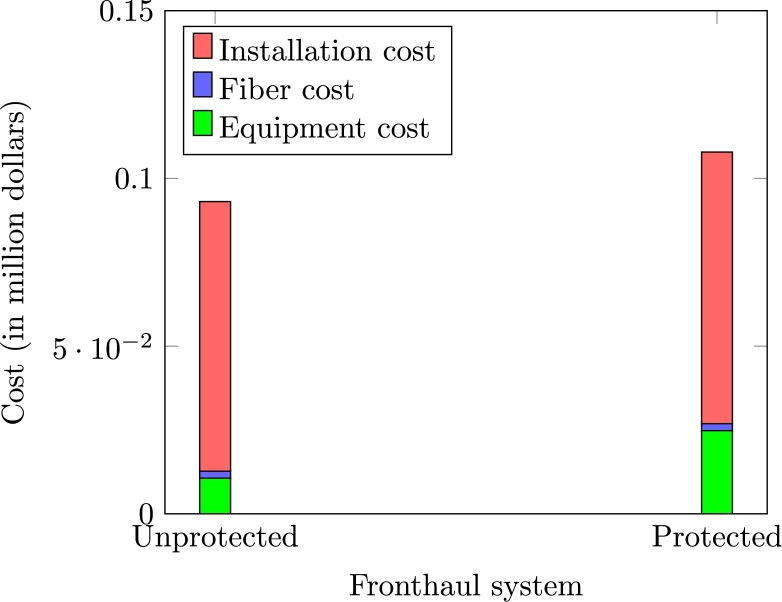
CAPEX Cost comparison of unprotected and protected fronthaul architectures.

It is evident from Fig. 5 that the major cost invested by the operator, in both protected and unprotected architecture, is on the deployment of fiber digging and trenches at FF and in the DF section. Fig. 5 also indicates that the deployment of dual-port OLT to support ring architecture at FF and the FSO link to protect DF increases the CAPEX cost of protected architecture. However, it brings substantial redundancy in the overall system with only 15% additional CAPEX cost. The additional protection cost not only saves the system from multiple types of failures, especially in dense urban localities, but also brings four nines reliability to the system.

## 5 Performance analysis

Here, in this section, we will analyze the transmission performance of the proposed protection model along with the optical power budget analysis to further verify the system's feasibility.

### 5.1 Performance analysis of the proposed protection scheme

The performance analysis of the proposed protection path has been carried out using the Opti system 17 simulation tool. We will analyze the Bit Error Rate (BER) versus the received optical power of the downstream channel for the DF section where we have installed the FSO link in a point-to-point topology. We have only considered the FSO performance as the FSO channel suffers from atmospheric turbulence, which is more pronounced in foggy conditions. Therefore, we will analyze its performance in different atmospheric turbulence. The different parameters used in the simulation are reported in Table 9, which includes all the losses of the FSO channel.

**Table 9:** Simulation parameters.

Parameter	Range
Fiber Length	10 km
TX Wavelength	1550 nm
FSO Link Distance	1 km
Data Rate/Wavelength	1.25 Gb/s
Transmitted Optical Power	10 MW
Beam Type	Narrow Beam
PIN Responsivity	0.56 A/W
Filter	Low Pass Bessel
Transmitter Aperture	10 cm
Receiver Aperture	10 cm
Transmitter Efficiency	0.75
Receiver Efficiency	0.8
Turbulence Parameter	Weak, Moderate, Strong
AWG Insertion Loss	3 dB
Atmospheric attenuation	1 dB/km
Feeder Fiber connector/ Splices Loss	0.07 dB
Refractive index structure parameter (Strong)	5 × 10^−12^*m*^−2/3^
Refractive index structure parameter (Moderate)	5 × 10^−14^*m*^−2/3^
Refractive index structure parameter (Weak)	5 × 10^−16^*m*^−2/3^
Number Of FSO Links	32

The designing of FSO transmission depends upon two factors, i.e., uncontrollable and controllable. The uncontrollable factor is weather dependent, while controllable factors depend upon the designing feature of FSO modules, such as the aperture size, the divergence angle and the link length of the FSO system. The FSO operators normally use large laser power to overcome these limitations (controllable and uncontrollable). However, large laser power is dangerous for human health; therefore, we have used an operating wavelength of 1550 nm in the simulation work. The transmitted power of 1550 nm wavelength does not affect human health, as it can be transparently coupled to single-mode fiber. In the simulation, the deployed FSO links are working on a LOS. Therefore, the FSO channel (*h*) is a function of three factors, i.e., $h=h_{P}\times h_{a}\times h_{g}$, where $h_{p}$ is the path loss that measures the degradation of the FSO beam due to fog, haze, rain and snow. The $h_{a}$ is the turbulence loss, i.e., scintillation occurs due to the change of refractive index of medium or atmosphere, and $h_{g}$ is the geometric loss depending upon the number of factors such as the FSO link length, the diameter of the transmitter/receiver apertures and laser beam divergence. The transmitter and receiver apertures are specified by the FSO components manufacturers, whereas the system designers set the divergence angle and link length.

To evaluate the performance of the protection path, as schemed in Fig. 3, we have arbitrarily chosen wavelengths located at the frequency range, i.e., $f_{n}=f_{i}+nf_{RF}$ where n=0 to 31, these wavelengths filtered out by using a 1:N de-multiplexer, where the value of *N* maybe 16, 24 or 32. From a set of 16, 24, or 32 wavelengths, each wavelength is modulated for the downstream data signal with Non-Return to Zero - On-Off Keying (NRZ-OOK) format at the rate of 1.25 Gb/s (this data rate is the requirement for a split option 6 or 7 as per 3GPP standard). The major advantage of using the NRZ-OOK format is that it occupies less bandwidth with sufficient DC balancing. As a result, the line coding or scrambling technique is not needed in the system (both techniques are used to maintain the necessary DC balancing). The *N* numbers of NRZ-OOK modulated optical signals are again fed to N×1 WDM-MUX. The combined signal is transmitted over single-mode FF after suitable optical amplification through $2^{nd}$ OA (length of FF is set at 10 km). The multiplexed signal again splits into *N* separate wavelengths at the RN location (1:N) through AWG.

The AWG will further pass each wavelength to the ONU through the FSO link. We have considered that all small cells are geographically located in 1 km. Therefore, the maximum length of DF/FSO from RN to ONU will be 1 km. On the ONU side, we have used the positive intrinsic negative (PIN) diode for photodetection compared to the avalanche photodiode (APD). The APD has a much larger gain as it offers better sensitivity than the PIN photodiode. However, APD has serious drawbacks, requiring a high operating voltage to provide a strong electric field. This problem can be addressed and compensated but at the expense of an increase in the network's cost. A PIN photodiode followed by an electronic amplifier could also provide good sensitivity with a low-cost solution.

#### 5.1.1 Simulation results

**Figure 6:**
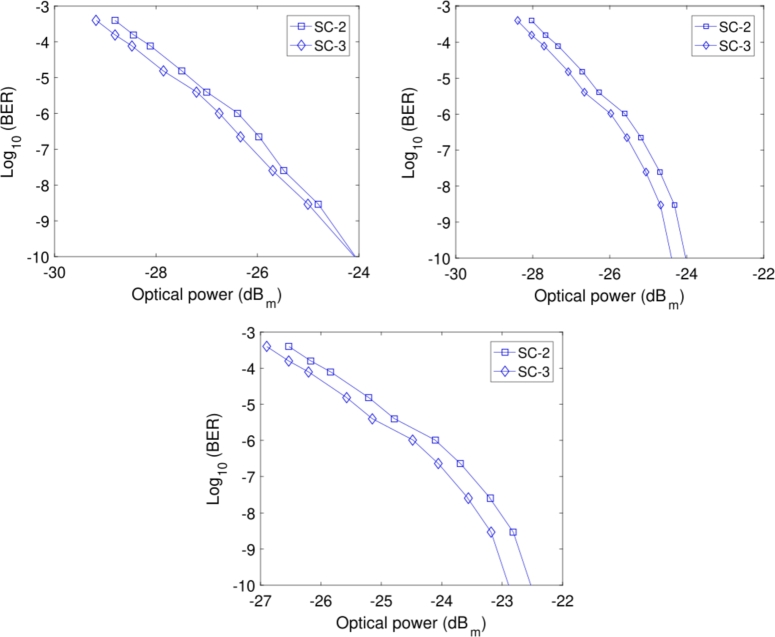
BER Analysis on FSO link in (top left) weak turbulence (top right) moderate turbulence (bottom) strong turbulence.

To analyze the effect of turbulence regimes, i.e., weak, moderate and strong, on FSO transmission, we have arbitrarily selected the small cells 2 and 3 to show the effects of different turbulence regimes on receiver sensitivity. It may be observed from Fig. 6(a) that the values of receiver sensitivity of small cells 2 and 3 are around −24.8 dBm and −24.5 dBm, respectively, for weak turbulence, i.e., $C_{n}^{2}=5\times 10^{-16}$. Similarly, Fig. 6(b) shows that the values of receiver sensitivity of small cells 2 and 3 are around −24.5 dBm and −24 dBm, respectively for moderate turbulence, i.e., $C_{n}^{2}=5\times 10^{-14}$. Finally, it may be observed from Fig. 6(c) that the values of the receiver sensitivity of small cell 2 and 3 become equal to −23 dBm and −22.7 dBm, respectively, for strong turbulence, i.e., $C_{n}^{2}=5\times 10^{-12}$. The results show that the overall receiver sensitivities of small cells are degraded when the turbulence increases by increasing the refractive index structure parameter $C_{n}^{2}$ from weak turbulence to strong turbulence. A small variation between values of receiver sensitivity of small cells in different turbulence is observed, which may be ignored. However, the overall BER performance is very good, which proves that the FSO link is also very robust and can provide a suitable alternative to the distribution fiber-based link in case of a fault.

### 5.2 Optical power budget analysis

The study of power budgeting or attenuation allowance is a significant tool to measure the performance of any telecommunication network, as it defines the network span, the total number of users the system can accommodate and the maximum data rate the system can support. In power budget analysis, we will identify the network component where power can drop or increase due to losses or component gain. In short, we will calculate all gains and losses of the proposed protected UDWDM PON from OLT-RN-FSO-ONU, i.e., at the PIN photodiode. The study's objective is to ensure enough power is reached from OLT to ONU or the power available above the receiver's sensitivity so that the fronthaul/backhaul system can work efficiently.

The overall optical power margin of the protected system can be calculated through the relation defined by Eq. (13). For the designed architecture, we have set the system's safety margin (SM) at 3 dB; normally, the operator considers an SM value above 1.7 dB for error-free transmission. Whereas the launched power from the laser source is set at 0 dB, the AWG port or splitting ratio is 32, the length of FF is considered 10 km, while the length of the FSO link is assumed maximum up to 1 km, and the receiver sensitivity is set as −34 dB. Table 10 lists the theoretical losses and gains of all the components used in the system.

(13) $$P_{M}=P_{B}+G-L_{MP}-S_{M}\geq \;\text{Receiver sensitivity}$$

$$L_{MP}=\sum L_{MUX}-L_{DeMUX}-L_{FF}(\alpha )-N\times L_{C}-L_{AWG}-L_{FSO}-L_{OS}$$

$$G={\text{EDFA}}_{1}+{\text{EDFA}}_{2}$$

where $P_{M}$ is the optical power margin, $P_{B}$ is the total launched optical power transmitted by the OLT laser and *G* is the total gain of the system. The $L_{MP}$ is the total power loss of the protected path, i.e., of all the passive optical components placed in the central office, ODN, and at the ONU. The $L_{Mux}$ and $L_{DeMUX}$ represent the losses that occur in Mux and DeMux. The $L_{FF}$ is the loss that occurs in the system due to the FF, which is the function of attenuation constant/km (*α*) and the product of the length of FF. The $L_{C}$ represents the losses that occur by the connector and splices, where *N* represents the total number of connectors /splices included in the system. The $L_{AWG}$ is the insertion loss in the system due to the AWG port. The term $L_{FSO}$ is the combined loss of the FSO system, which is the function of the FSO transceivers/modules and the product of atmospheric attenuation/km, while $L_{OS}$ shows the loss occurring due to optical switch, which is installed at the ONU.

**Table 10:** Optical power budget analysis.

Component	dB
MUX	−12
DeMUX	−12
AWG	−3
EDFA	50 (i.e., 25×2)
Fiber Attenuation/km	2 (0.2/km over 10 km of SMF)
FSO Transceiver (internal loss of the FSO equipment)	0 (supposed to be 0 dB for an ideal case)
FSO atmospheric attenuation	−3
Optical Switches	−0.5
Connector losses/splices losses	0.07 (i.e., 2×0.035)
Minimum Receiver Sensitivity (NRZ-OOK)	−34
**Power Budget (dB)**	**14.43**

By combining all the losses and gains of the system from Table 10 and putting in Eq. (13), the total optical powers available at the input of PIN of the proposed architecture is 14.43 dB which is still positive. Therefore we can say that the available power budget of the system is reasonable and can support the fronthaul/backhaul connectivity to the given numbers of small cells.

$$P_{M}=G(50)-\sum L(32.57)-S_{M}(-3)>-34\text{ dB}$$

$$P_{M}=14.43\text{ dB}$$

## 6 Conclusion

In this paper, we proposed a cost-effective, reliable, and efficient protection scheme for the 5G fronthaul/backhaul segment through UDWDM PON. We observed from the analysis that the proposed scheme provided system reliability up to four nines, i.e., 99.9946% with only 15% additional CAPEX cost. It is also evident from our analysis that the deployment of ring-based protection at the FF and FSO-based protection at the DF prevents the system from unnecessary outage and decreases the cost of protection. It may be observed from CAPEX costs that the system deployment cost proportionally depends upon the digging, trenching, and pumping of fiber, so giving protection to the new sites with a wireless approach can decrease the protection cost substantially compared to complete fiber-based protection architecture. Moreover, the efficacy of the FSO system, i.e., its vulnerability to atmospheric attenuation, is also analyzed from the BER versus received optical power plot and through link budget analysis. Therefore, we can suggest that the proposed UDWDM PON-based architecture with the ring-based protection at FF and FSO-based protection scheme at DF is feasible, especially for deploying small cells in dense urban areas. The proposed work may be extended by considering the higher split ratios like 1:64 and 1:128. Moreover, efficient modulation formats such as 64-QAM and PAM-4 may make the design more spectrally efficient.

## Author contribution statement

Syed Saeed Jaffer, Ashiq Hussain, Muhammad Ali Qureshi, Yousaf Khan, Jawad Mirza, Khurram Karim Qureshi, and Muhammad Mahmood Ali: Conceived and designed the experiments; Performed the experiments; Analyzed and interpreted the data; Contributed reagents, materials, analysis tools or data; Wrote the paper.

## Funding statement

This work was partially supported by the center of communication systems and sensing at 10.13039/501100004055KFUPM [INCS2206].

## Declaration of Competing Interest

The authors declare no competing interests.

## Data Availability

Data will be made available on request.

## Footnotes

1 https://www.ericsson.com/en/reports-and-papers/mobility-report/dataforecasts/mobile-traffic-forecast.

2 http://www.OEQUEST.com.
